# Effectiveness of genetic feedback on alcohol metabolism to reduce alcohol consumption in young adults: an open-label randomized controlled trial

**DOI:** 10.1186/s12916-024-03422-y

**Published:** 2024-05-20

**Authors:** Yukiko Owaki, Hisashi Yoshimoto, Go Saito, Shohei Dobashi, Satoshi Kushio, Akihiro Nakamura, Takahiro Goto, Yusuke Togo, Kazumasa Mori, Hideki Hokazono

**Affiliations:** 1 Department of Nursing, School of Nursing, Gunma Prefectural College of Health Sciences, Maebashi, Japan; 2 Research and Development Center for Lifestyle Innovation, University of Tsukuba, Tsukuba, Japan; 3 Department of Family Medicine, General Practice and Community Health, Institute of Medicine, University of Tsukuba, Tsukuba, Japan; 4 Department of Primary Care and Medical Education, Graduate School of Comprehensive Human Sciences, University of Tsukuba, Tsukuba, Japan; 5 Sanwa Laboratory, Sanwa Shurui Company, Usa, Japan

**Keywords:** Excessive drinking, *ALDH2*, *ADH1B*, Gene polymorphisms, Japanese, College students, Transtheoretical model, Young adults, Randomized controlled trial

## Abstract

**Background:**

It is unclear whether brief interventions using the combined classification of alcohol-metabolizing enzymes aldehyde dehydrogenase 2 (*ALDH2*) and alcohol dehydrogenase 1B (*ADH1B*) together with behavioral changes in alcohol use can reduce excessive alcohol consumption. This study aimed to examine the effects of a brief intervention based on the screening of *ALDH2* and *ADH1B* gene polymorphisms on alcohol consumption in Japanese young adults.

**Methods:**

In this open-label randomized controlled trial, we enrolled adults aged 20–30 years who had excessive drinking behavior (average amount of alcohol consumed: men, ≥  4 drinks/per day and women, ≥  2 drinks/per day; 1 drink = 10 g of pure alcohol equivalent). Participants were randomized into intervention or control group using a simple random number table. The intervention group underwent saliva-based genotyping of alcohol-metabolizing enzymes (*ALDH2* and *ADH1B*), which were classified into five types. A 30-min in-person or online educational counseling was conducted approximately 1 month later based on genotyping test results and their own drinking records. The control group received traditional alcohol education. Average daily alcohol consumption was calculated based on the drinking diary, which was recorded at baseline and at 3 and 6 months of follow-up. The primary endpoint was average daily alcohol consumption, and the secondary endpoints were the alcohol-use disorder identification test for consumption (AUDIT-C) score and behavioral modification stages assessed using a transtheoretical model.

**Results:**

Participants were allocated to the intervention (*n* = 100) and control (*n* = 96) groups using simple randomization. Overall, 28 (29.2%) participants in the control group and 21 (21.0%) in the intervention group did not complete the follow-up. Average alcohol consumption decreased significantly from baseline to 3 and 6 months in the intervention group but not in the control group. The reduction from baseline alcohol consumption values and AUDIT-C score at 3 months were greater in the intervention group than in the control group (*p* < 0.001). In addition, the behavioral modification stages were significantly changed by the intervention (*p* < 0.001).

**Conclusions:**

Genetic testing for alcohol-metabolizing enzymes and health guidance on type-specific excessive drinking may be useful for reducing sustained average alcohol consumption associated with behavioral modification.

**Trial registration:**

R000050379, UMIN000044148, Registered on June 1, 2021.

**Supplementary Information:**

The online version contains supplementary material available at 10.1186/s12916-024-03422-y.

## Background

Excessive alcohol consumption causes various health problems, and many studies on countermeasures have been performed [1–11]. Abnormal alcohol consumption not only causes acute alcohol poisoning but also results in dangerous behaviors in the intoxicated state, such as those leading to injuries, rape, and suicide [1, 2]. Young adulthood is especially recognized as a significant risk period for increased levels of alcohol abuse behaviors such as binge and high-intensity drinking [4]. Moreover, the occurrence of high levels of alcohol consumption with other transdiagnostic factors early in life can lead to alcohol-use disorder in later stages of life [5, 12], suggesting the importance of designing public health strategies to prevent high-risk levels of alcohol consumption in young adults.

Young people with alcohol-use disorders have been reported to have low rates of health literacy [13]. Consequently, the effects of education on drinking behavior (i.e., brief interventions targeting young people) on alcohol consumption have recently been examined. Brief interventions for preventing high-risk levels of alcohol-use behavior include counseling sessions, which typically consist of face-to-face counseling sessions delivered by a trained interventionist (e.g., physicians, psychologists, nurses, or social workers), designed to increase people’s awareness of their alcohol use and its consequences [14]. Previous meta-analytic evidence has shown that brief alcohol interventions are efficacious in reducing alcohol use among young adults [15, 16]; nonetheless, the effects can be modest in magnitude [4]. Therefore, an effective method other than brief interventions or methods that promote the effectiveness of brief interventions should be established.

Focusing on the genetic polymorphisms of alcohol-metabolizing enzymes, East Asia, which accounts for approximately 22% of the world’s population, has specific genetic polymorphisms in alcohol-metabolizing enzymes aldehyde dehydrogenase 2 (*ALDH2*) and alcohol dehydrogenase 1 B (*ADH1B*) [6, 7]. Functional variants in *ALDH2* and *ADH1B* make the population less tolerant to alcohol consumption [8]. Recent studies have indicated that genetic polymorphisms of alcohol-metabolizing enzymes, including *ALDH2* and *ADH1B*, are associated with various alcohol-related diseases such as cancer, heart disease, and liver disease [7, 9–11]. Understanding an individual’s alcohol-metabolizing enzyme genotype and implementing preventive behaviors accordingly may contribute to solving alcohol-related health problems.

Hendershot et al. screened genetic polymorphisms of *ALDH2* in 200 Asian-American young adults and provided health risk information on alcohol-related cancer and addiction along with the test results [17]. The results showed that participants with low *ALDH2* activity (ALDH2*1/*2 genotypes) significantly reduced the frequency and amount of alcohol consumption 30 days after the intervention [17]. Furthermore, Ishikawa showed that the average daily alcohol consumption of the intervention group that received health guidance in addition to information on *ALDH2* polymorphisms was significantly lower than that of the control group that did not receive genetic information 6 months after the intervention [18]. To the best of our knowledge, no intervention has been designed based on genetic information feedback of *ADH1B* polymorphism, which is also associated with alcohol-related diseases, similar to those associated with *ALDH2*. *ALDH2* and *ADH1B* genetic polymorphisms are independent of each other, and both genotypes may be dysfunctional or only one may degenerate. Hence, genetic polymorphism testing for both genotypes can further subdivide the classification of alcohol tolerance, and brief interventions based on the subdivisions may be useful as novel approaches to reduce alcohol consumption. In addition, a previous study has suggested that reduction in alcohol consumption due to a brief intervention using genetic information feedback was attributed to behavioral changes toward alcohol consumption [19]; however, no study has investigated the effects of brief interventions on alcohol-use behavior in relation to alterations in behavioral modification stages.

Based on this background, we developed a method to classify five major types of drinking habits based on *ALDH2* and *ADH1B* gene test results and implement a simple intervention based on these five types (Table 1) [20]. Therefore, in this study, we aimed to examine the effects of a brief intervention based on the screening of *ALDH2* and *ADH1B* gene polymorphism on alcohol consumption (which is called screening and brief intervention [SBI]) in Japanese young adults aged 20–30 years with excessive drinking habits in relation to behavioral change in a transtheoretical model (TTM) [21].

**Table 1:** Composition of the five genotypes of alcohol-metabolizing enzymes and associated health risks [20, 22]

*Aldehyde dehydrogenase*	*Alcohol dehydrogenase*	Classification by type (%)^a^	Comments on constitution and health risks
*ALDH2* *1/*1 (activity)	*ADH1B* *1/*1 (low activity)	A (3%)	Highest risk of alcohol addiction
	*ADH1B* *1/*2 (activity)	B (50%)	Decomposition of alcohol and acetaldehyde is fast. Care should be taken not to drink too much
	*ADH1B* *2/*2 ( activity)		
*ALDH2* *1/*2 (low activity)	ADH1B *1/*1	C (3%)	The decomposition of alcohol and acetaldehyde is slow, and the cancer risk is high, owing to acetaldehyde
	ADH1B *1/*2	D (40%)	Face turns reddish immediately. Nausea and other discomfort can occur. Symptoms and health problems are likely to occur
	ADH1B *2/*2		
*ALDH2* *2/*2 (Inactive)	ADH1B *1/*1	E (4%)	Drinking is intolerable because aldehyde cannot be decomposed Very small amounts of alcohol cause unpleasant symptoms such as hot flushes, drowsiness, palpitations, and nausea Even a small amount of alcohol poses a risk of acute alcohol intoxication
	ADH1B *1/*2		
	ADH1B *2/*2		

^a^Percentage of Japanese

## Methods

This study was an open-label, randomized controlled trial and was conducted at University of Tsukuba according to the study protocol published in 2022 [22].

### Participants

The inclusion criteria were as follows: (i) undergraduate and postgraduate students, faculty, and staff of the University of Tsukuba; (ii) those with excessive drinking habits of pure alcohol consumption (average amount of alcohol consumed: men, ≥  4 drinks/per day and women, ≥  2 drinks/per day; 1 drink = 10 g of pure alcohol equivalent), which increases the risk of lifestyle-related diseases according to Japanese standards; (iii) those aged 20–30 years; and (iv) those with good health with no health interference in their daily life, studies, or employment and with no history of prior or current illness. The exclusion criteria were as follows: (i) those who did not wish to disclose their alcohol constitution test results and (ii) those with difficulty communicating in Japanese.

Participation was terminated if the participants opted to discontinue at any point in the study.

### Informed consent and randomization

Potential study participants, recruited through poster advertisement and snowball sampling at the university, received a written explanation of the study and agreed to participate by signing a consent form. Participants were assigned to the intervention (individual intervention on drinking habits was provided; at enrollment, participants underwent genetic testing for alcohol-metabolizing enzymes using saliva, and approximately 1 month later, they received a brief intervention that included the test results and intervention instructions) or control (no constitutional testing was conducted during the study period, and only conventional educational materials were provided) group.

### Measures

A self-administered questionnaire anonymized by ID number was used to investigate the following: basic attributes (age, sex, main field of study, living with family, employment status, participation in clubs, hobbies, and activities) and outcome measures (primary outcome: average daily alcohol consumption; secondary outcomes: stages of change, Alcohol Use Disorders Identification Test for consumption [AUDIT-C], and AUDIT as screening tests for excessive alcohol consumption) [23]. The researcher (primarily the first author) sent the questionnaire and the drinking calendar to the participants via e-mail. The participants were requested to fill out the questionnaires and return them within 1 week of receiving them. If the filled questionnaires were not returned, an optional reminder e-mail was sent within 1 month. Data were collected from the returned attachments.

The intervention group was surveyed 1 month after the intervention and 3 and 6 months after obtaining consent for participation in this study. Data from a calendar that recorded the nature and amount of alcohol consumption were collected concurrently. In the control group, data from a calendar that simultaneously recorded alcohol consumption and quantity were collected at 1, 3, and 6 months after the participants provided consent for participation in this study. Initially, we planned to use the values at 1 month after the intervention for comparisons between the groups, but the timing of the observation was different from that of the control group due to a slight delay in the return of the genotype test results. Therefore, only the data at baseline and at 3 and 6 months were used for the analysis in this study.

### Primary outcome

The primary outcome was the average daily alcohol consumption (g of pure alcohol equivalent) of participants, which was calculated using a generally accepted formula and the following procedure.

A drinking calendar showed drinking examples that were similar to those in the questionnaire, and participants were requested to record the content (type and concentration of alcohol) and amount of alcohol consumed on each drinking day during a 6-month period. From the calendar records, the net alcohol consumed (g) was calculated using the following generally accepted formula: net alcohol content (g) = volume of liquor (mL) × degree or %/100 × 0.8 (specific gravity). This formula was used to calculate the net alcohol content for each study period, which was divided by the number of drinking days, to calculate the average daily alcohol consumption (g of net alcohol).

The following examples are included in the alcohol consumption questionnaire.

Conversion for alcoholic beverages was as follows: sake: 15% alcohol content, 180 mL = 2 drinks; beer: 5% alcohol content, 500 mL [medium bottle, medium mug, or canned beer] = 2 drinks; whiskey and brandy: 43% alcohol by volume, 60 mL [double] = 2 drinks; shochu: 25% alcohol by volume, 100 mL [half cup] = 2 drinks; canned cocktail: 7% alcohol by volume, 350 mL = 2 drinks; cocktails, fruity tastes, etc.: 5% alcohol by volume, 350 mL [can] = 1.5 drinks, 500 mL [can] = 2 drinks; wine: 12% alcohol by volume, 150 mL [glass] = 1.5 drinks; and umeshu: 12% alcohol by volume, 90 mL [small glass] = 1 drink. In the survey, respondents were asked to indicate the average number of drinks they consumed per day (1 drink = 10 g pure alcohol) on their drinking days during the past month.

### Secondary outcomes

The AUDIT and AUDIT-C were assessed. The AUDIT (10 items, 40-point scale) was developed by the World Health Organization (WHO) as a screening method for excessive drinking. The question items and response options for AUDIT are shown in Additional file 1: Table S1; they are rated by summing the number of relevant options for the question items. The validity and effectiveness of the Japanese version of the AUDIT have been validated by Hiro et al. [24]. At present, in terms of screening and brief intervention for excessive drinking in Japan, an AUDIT score of 7 or less is considered low-risk drinking, whereas an AUDIT score of 8 or more suggests excessive drinking [25]. The AUDIT-C is a simple screening test (12-point scale) consisting of the AUDIT Questions 1–3. In Japan, a score of ≥  6 for men and ≥  4 for women is considered excessive drinking [25].

Moreover, the five stages of change in drinking behavior include precontemplation, contemplation, preparation, action, and maintenance [21]. The stages of change model is a theory used to understand current motivational perceptions and stages of behavior change to provide more appropriate health guidance. Specifically, the survey required responses from the following five options: “precontemplation” with no intention to change behavior within 6 months, “contemplation” with intention to change behavior within 6 months, “preparation” with intention to change behavior within 1 month, “action” with less than 6 months since behavioral change, and “maintenance” with more than 6 months since behavioral change.

### Alcohol-metabolizing enzymes related to genotype testing and five types of classification

All participants in the intervention group underwent genetic testing for alcohol-metabolizing enzymes and received a report of their results. In the control group, those who requested testing were tested at the end of the study period, and they received their results.

Those with type A had low *ADH1B* activity and high *ALDH2* activity, resulting in long-lasting effects of alcohol and rapid degradation of acetaldehyde. Therefore, those with type A had the highest preference for alcohol and were most likely to become dependent. Those with type B had both *ADH1B* and *ALDH2* activities and were the most susceptible to drinking among the five types; therefore, such individuals needed to be careful not to drink excessively. Those with type C had slow decomposition of alcohol and acetaldehyde; therefore, it was difficult for them to be aware of acetaldehyde generation and discomfort, but they had a risk of carcinogenesis. Those with type D showed rapid alcohol decomposition and slow acetaldehyde decomposition. Therefore, their blood concentration of acetaldehyde increases, causing immediate facial redness, nausea, and other unpleasant symptoms and acetaldehyde-related health problems. Those with type E had inactive *ALDH2*, could not decompose acetaldehyde, and were at risk of acute alcohol intoxication, even with small amounts of alcohol.

### Intervention using genotype test information

Individual interventions using genotype results were conducted at University of Tsukuba according to the study protocol [22]. Both the intervention and control groups lived a normal life with no special restrictions. The intervention was based on individual test results, baseline questionnaire data, drinking calendar records, and health guidance materials and included conventional teaching materials [26]. The common and main content of the intervention for the five types of alcohol-metabolizing enzyme genotyping concerned how to deal with alcohol according to the participants’ alcohol consumption, mechanism of alcohol absorption, and alcohol intake in nutrition, eating, and drinking. The specifics are as follows:

- Alcohol constitution of the participants (from A to E and characteristics of each constitution type)—alcohol degradation mechanisms, metabolic enzyme functions, and constitution based on the combination of alcohol-metabolizing enzymes
- Disease risks associated with alcohol consumption and points to note regarding alcohol constitution (comments were provided by the five constitution types)
- How to deal with alcohol according to participants’ alcohol constitution, how to plan drinking/eating in terms of nutrition, and how to slow down alcohol metabolism
- Drinking habit screening test (AUDIT-C)
- How to drink appropriately and rest days
- Inappropriate drinking (including drinking during pregnancy and lactation, drunk driving, alcohol harassment, and habitual heavy drinking)

Interventions were primarily conducted by the first author who is a licensed nurse. Before the intervention, two main physicians in charge of the outpatient alcohol consumption reduction clinic at University of Tsukuba Hospital trained and advised the researcher on the brief intervention.

Of note, face-to-face (*n* = 18) or online interventions (*n* = 82) were conducted according to the participants’ needs as the study was conducted during the coronavirus disease 2019 pandemic and infection control measures were required.

### Conventional teaching materials for the control group

In the control group, conventional educational materials (an alcohol handbook for college students) [26] were provided to the participants to read during the consent process.

The main components of the conventional material [26] are as follows:

- Recollections of the loss of a child or parent due to a fatal accident or illness caused by drinking
- Current drinking styles of college students (results of the Drinking Attitudes Survey)
- Conversion of the amount of alcohol consumed on a given drinking day using the drinking amount conversion chart and confirmation of current drinking amount using the drinking amount ranking chart (males and females)
- Stages from tipsiness to death due to acute alcohol intoxication
- Monitoring and dealing with binge drinking, alcohol, and harassment
- Devices and specific examples of appropriate drinking at drinking parties
- Knowledge about alcohol (alcohol-related problems: such as violence, depression, sleep-related problems, family and workplace problems, and mental and physical illness)

### Sample size

The total sample size of this study was 199, calculated with an effect size of 0.2, an alpha error of 0.05, and a power of 0.8 using a power analysis software (G* Power3.1, University of Kiel, Germany) [27], as reported previously [17, 28]. In total, 200 participants (100 in the intervention and control groups) were enrolled.

### Statistical analysis

The available data for all participants were included according to the original allocation in an intention-to-treat analysis. The unsubmitted data of participants who dropped out were treated as missing data. Statistical analysis for the continuous variable scale was performed after confirmation of normality using the Shapiro–Wilk test. None of the continuous variables was normally distributed. For the baseline characteristics of age, AUDIT-C, AUDIT, average number of drinks, and average daily alcohol consumption, Wilcoxon’s rank-sum test was used for comparisons between the control and intervention groups. For the baseline characteristics of distribution of females, carrier status, status of employment, circle and hobby activities, and status of living (with family or not), chi-square test was used for comparisons between the groups. Moreover, the differences in the distribution of classifications based on alcohol-metabolizing enzyme genotype testing and behavioral alteration stage between the groups were compared using Fisher’s exact probability test.

The time-course changes in outcomes (e.g., alcohol consumption and AUDIT-C) were compared between the two groups using mainly two-way (group × time) analysis of variance (ANOVA). However, as many dropouts were unexpectedly observed in the current study, we judged that two-way ANOVA was not suitable for comparison between the groups for which randomization might not be preserved. Therefore, the analytical method was divided into two viewpoints as follows: time-series comparisons within the same group and group comparisons at the same time point, in which all available data for each analysis were used. Thus, the time-course changes in alcohol consumption and AUDIT-C score regarding the values at baseline versus at 3 and 6 months of follow-up and the values at 3 versus 6 months were compared using Wilcoxon’s signed-rank test with Bonferroni correction within the same group, respectively. For group comparisons at the same time point, the changes from baseline alcohol consumption values and AUDIT-C scores were compared at 3 and 6 months.

The statistical significance level was set at less than 5% (*p* < 0.05), and Stata/SE 18.0 (Stata Corp., USA) was used for the analysis. All graphs were generated using Prism version 9.5.1. (GraphPad, USA).

### Ethics and dissemination

This study was approved by the Ethics Committee of the University of Tsukuba (Human Genome and Genetic Analysis Research). The recruitment of participants began in July 2021, and the survey was completed in June 2023.

The researchers strictly maintained a list of participants matched by ID numbers and managed the data so that participant names and genotypic constitution information were not directly linked. Data entry and analysis were performed by the first author using a personal computer with security measures, and the results were confirmed by the co-researcher.

This study complied with the Ethical Guidelines for Human Genome and Genetic Analysis Research issued by the Ministry of Health, Labor, and Welfare of Japan. Enrollment in the UMIN clinical trial was completed on June 1, 2021.

## Results

### Participants

**Fig. 1:**
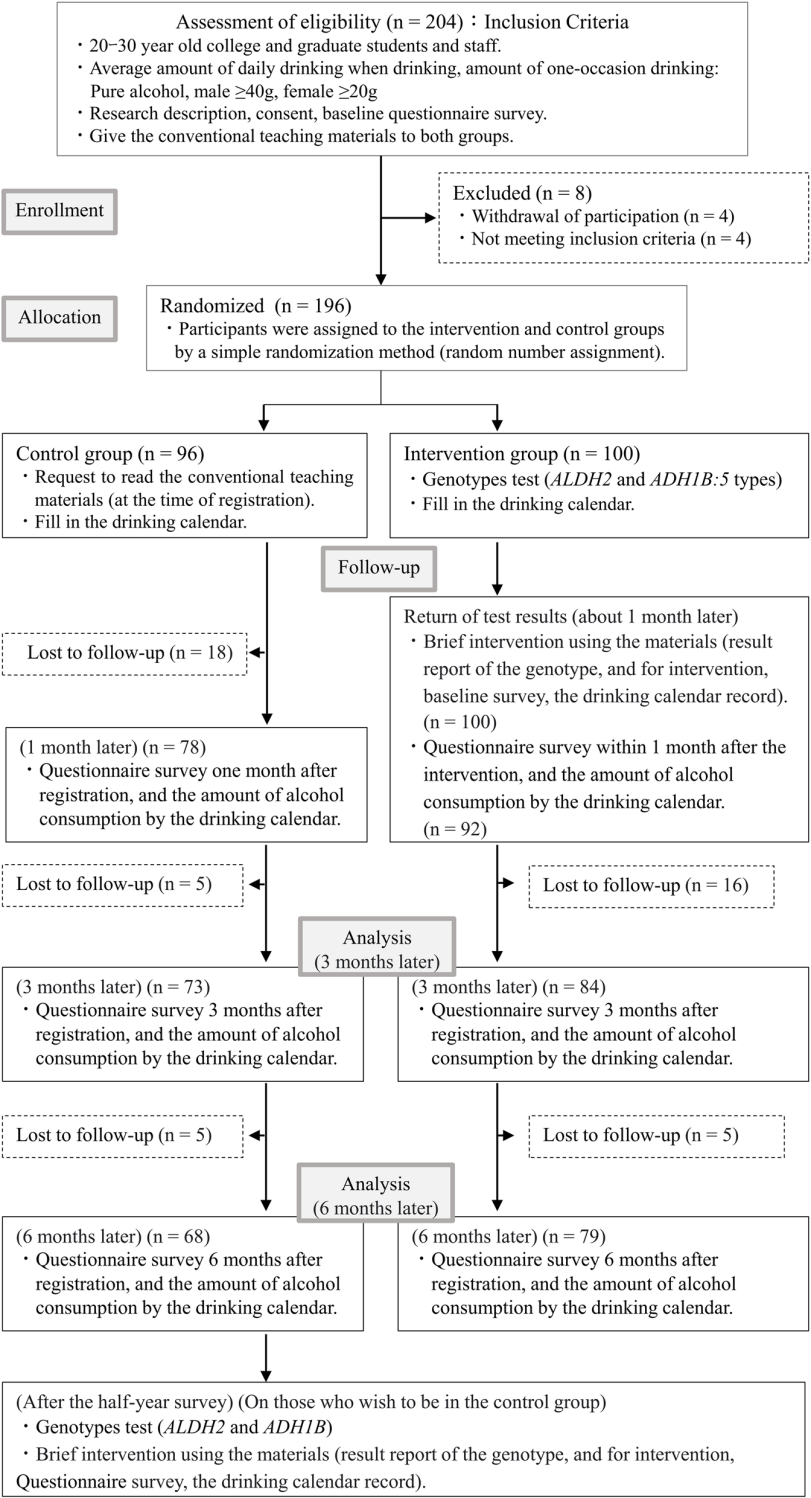
Consolidated Standards of Reporting Trials (CONSORT) flow chart

Figure 1 presents the Consolidated Standards of Reporting Trials flowchart for this study. Undergraduate and postgraduate students and university staff were invited to participate in the study between July 2021 and October 2022, and enrollment was completed by November 2022. The final follow-up was completed in June 2023. Consequently, 204 patients were enrolled in this study. Of these, four patients withdrew from this trial during the study period after enrollment (three withdrew because they were too busy to drink and one withdrew because a physician ordered abstinence from alcohol due to alcohol-related illness), and four who could not be contacted were excluded from the analysis. In total, 196 individuals were selected for this study. Participants in the intervention group were randomly allocated using a simple randomization method (intervention group, *n* = 100; control group, *n* = 96) and underwent the genotype test (*ALDH2* and *ADH1B*: 5 types) with consent at enrollment. Individual intervention was conducted after the test results were returned (approximately 1 month later). Finally, 28 (29.2%) participants in the control group and 21 (21.0%) in the intervention group did not complete follow-up assessments.

Table 2 shows the baseline characteristics (i.e., age, sex, social position, AUDIT, AUDIT-C score, average daily average number of drinks, and baseline average daily alcohol consumption per month [calculated in g pure alcohol equivalent as “total alcohol equivalent for each study period divided by the number of drinking days”]). No significant differences were observed between the control and intervention groups in terms of age, carrier status, employment, participation status in circle or hobby activities, current living status, proportion of classification based on alcohol-metabolizing enzyme genotype testing, AUDIT and AUDIT-C scores, number of drinks consumed, and alcohol consumption value. Unexpectedly, the proportions of men and women in the groups were significantly different between the control and intervention groups (*p* < 0.01).

**Table 2:** Baseline characteristics of the participants (*n* = 196)

Variable	Control (*n* = 96)		Intervention (*n* = 100)		*P*-value
	*n*	% or mean (median)	*n*	% or mean (median)	
Age (years)	96	22.4 (22.0)	100	22.7 (22.0)	0.319^a^
Female	64	66.7	47	47.0	0.005^b^
Carrier status					0.798^b^
Undergraduate student	63	65.6	61	61.0	
Graduate student	21	21.9	25	25.0	
Faculty and staff	12	12.5	14	14.0	
Employment					0.718^b^
Not working	13	13.5	15	15.0	
Part-time job	71	74.0	69	69.0	
University staffs, resident, teachers or part-time lecturers	12	12.5	16	16.0	
Circle and hobby activities					0.720^b^
Have	60	62.5	60	60.0	
Not	36	37.5	40	40.0	
Currently status of living					0.604^b^
Living with family	16	16.7	14	14.0	
Living alone	80	83.3	86	86.0	
Classification based on alcohol-metabolizing enzyme genotype testing					0.979^c^
A	4	4.2	5	5.0	
B	54	56.3	78	78.0	
C	1	1.0	1	1.0	
D	12	12.5	16	16.0	
E	0	0.0	0	0.0	
AUDIT	96	8.6 (8.0)	100	9.1 (7.0)	0.543^a^
AUDIT-C	96	5.6 (5.5)	100	5.8 (6.0)	0.443^a^
Average number of drinks	96	5.4 (5.0)	100	5.8 (5.2)	0.250^a^
Average daily alcohol consumption (g)	96	42.3 (39.6)	100	44.6 (38.8)	0.603^a^

Statistical comparisons between control and intervention groups using ^a^Wilcoxon’s rank-sum test, ^b^chi-square test, and ^c^Fisher’s exact probability test, respectively

### Time-course changes in alcohol consumption and AUDIT-C score

**Fig. 2:**
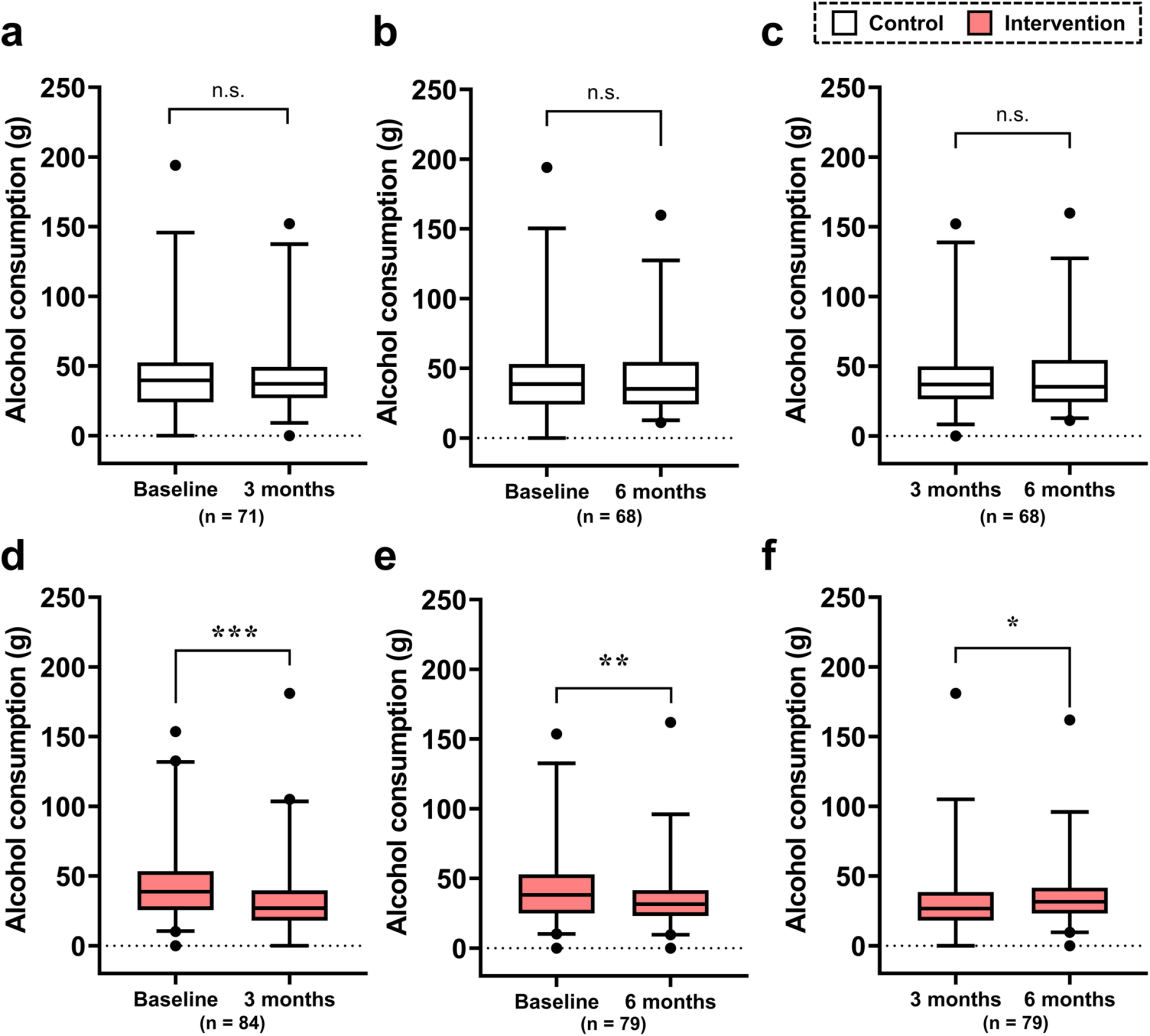
Time-course changes in alcohol consumption in the control and intervention groups. **a**–**c** The results for the control group. **d**–**f** The results for the intervention group. Baseline vs. 3 months (**a** and **d**), baseline vs. 6 months (**b** and **e**), and 3 vs. 6 months (**c** and **f**). The box represents the interquartile range (IQR), with the lower edge of the box indicating the 25th percentile and the upper edge indicating the 75th percentile. The whiskers extend from the box to the 2.5th and 97.5th percentiles. Wilcoxon’s signed-rank test was performed with the Bonferroni correction. Briefly, since time-series comparisons were conducted three times within the same group, the *p*-values calculated by Wilcoxon’s test were tripled, and those less than 0.05 were considered statistically significant. n.s., not significant; ***p < 0.001, **p < 0.01, and *p < 0.05

Figure 2 shows the time-course changes in alcohol consumption from baseline to 3 and 6 months and from 3 to 6 months. In the control group, no significant differences were observed between any of the time points (Fig. 2a–c). Alcohol consumption in the intervention group decreased from baseline to 3 and 6 months (both *p* < 0.01, Fig. 2d, e). However, it was significantly higher at 6 months than at 3 months (*p* < 0.05, Fig. 2f).

**Fig. 3:**
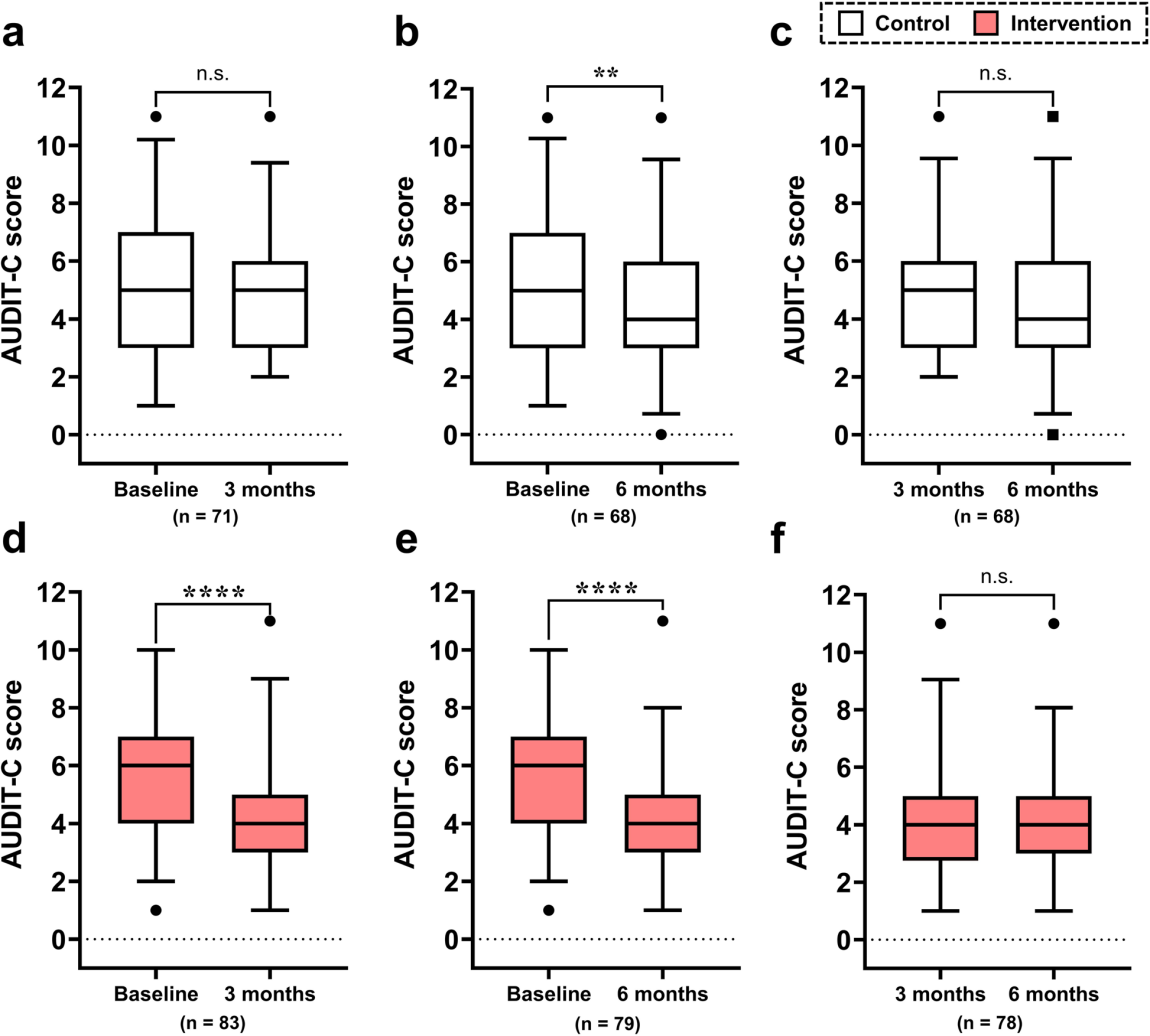
Time-course changes in AUDIT-C scores in the control and intervention groups. **a**–**c** The results for the control group. **d**–**f** The results for the intervention group. Baseline vs. 3 months (**a** and **d**), baseline vs. 6 months (**b** and **e**), and 3 vs. 6 months (**c** and **f**). The box represents the interquartile range (IQR), with the lower edge of the box indicating the 25th percentile and the upper edge indicating the 75th percentile. The whiskers extend from the box to the 2.5th and 97.5th percentiles. Wilcoxon’s signed-rank test was performed with the Bonferroni correction. Briefly, since time-series comparisons were conducted three times within the same group, the *p*-values calculated by Wilcoxon’s test were tripled, and those less than 0.05 were considered statistically significant. n.s., not significant; ****p < 0.0001, **p < 0.01

Figure 3 demonstrates the time-course changes in the AUDIT-C score from baseline to 3 and 6 months and from 3 to 6 months. In the control group, no significant differences were observed in AUDIT-C scores between baseline and 3 months and between 3 and 6 months (Fig. 3a, c), whereas those at 6 months were significantly decreased from baseline (*p* < 0.01, Fig. 3b). In the intervention group, the AUDIT-C score was significantly reduced from baseline to 3 and 6 months (both *p* < 0.001, Fig. 3d, e) but was not significantly different between 3 and 6 months (Fig. 3f).

### Differences in changes in alcohol consumption and AUDIT-C scores from baseline between the control and intervention groups

**Fig. 4:**
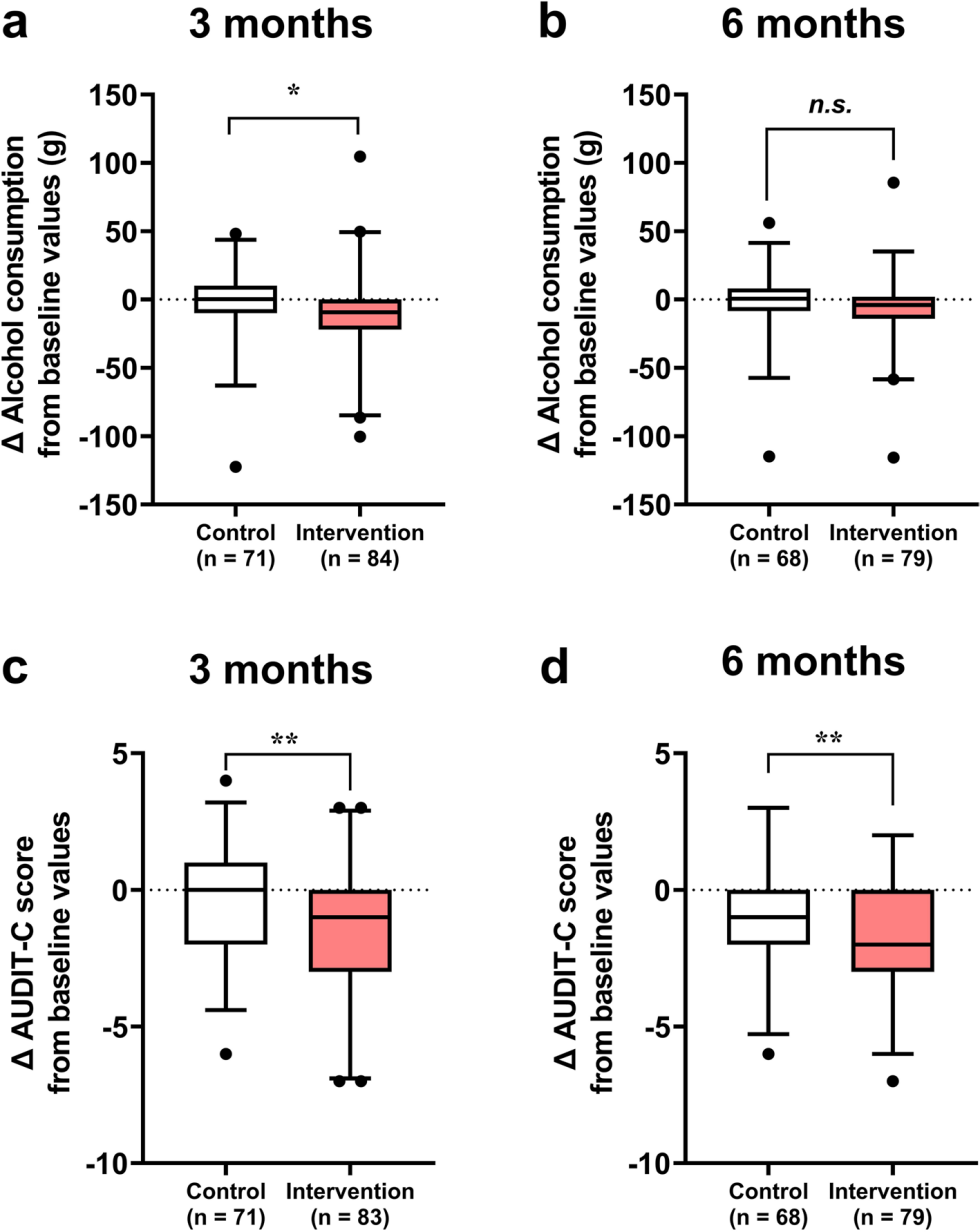
Changes in alcohol consumption and AUDIT-C scores from baseline between the control and intervention groups. **a**, **b** The changes in alcohol consumption from baseline values at 3- and 6-month follow-up, respectively. **c**, **d** The changes in the AUDIT-C score from baseline values at 3- and 6-month follow-up, respectively. The box represents the interquartile range (IQR), with the lower edge of the box indicating the 25th percentile and the upper edge indicating the 75th percentile. The whiskers extend from the box to the 2.5th and 97.5th percentiles. Statistical comparisons between the two groups in each panel were conducted using analysis of covariance (ANCOVA), with sex as a covariate. n.s., not significant; **p < 0.01, *p < 0.05

Unexpectedly, many dropouts were observed in the current study; thus, the changes in alcohol consumption and AUDIT-C scores from baseline were compared between the control and intervention groups at 3 and 6 months, respectively (Fig. 4). We also compared the baseline characteristics after eliminating dropouts, and significant differences in the proportion of men and women between the groups remained at 3 and 6 months (Additional file 1: Table S2 and S3). Since sex is assumed to have a significant effect on the change in alcohol consumption during alcohol reduction interventions [29], we compared the group differences in the changes in alcohol consumption and AUDIT-C score at 3 and 6 months using one-way analysis of covariance with sex as a covariate. Changes in alcohol consumption from baseline were significantly lower in the intervention group than in the control group at 3 months (*p* < 0.05, Fig. 4a) but not at 6 months (Fig. 4b). The changes in AUDIT-C scores from baseline were significantly lower in the intervention group than in the control group at both 3 and 6 months (*p* < 0.01, Fig. 4c; *p* < 0.01, Fig. 4d, respectively).

### Alteration in behavioral modification stage regarding alcohol-use behavior due to the brief intervention throughout the study period

The alteration in the behavioral modification stage regarding alcohol-use behavior due to the brief intervention throughout the study period is shown in Table 3.

**Table 3:** Alteration in behavioral modification stage distribution at baseline and 3- and 6-month follow-up

Time-course	Behavioral modification stage	Control		Intervention		*P*-value
		*n*	%	*n*	%	
Baseline						0.653
(control: *n* = 95; intervention: *n* = 100)	Precontemplation	85	89.5	86	86.0	
	Contemplation	5	5.3	4	4.0	
	Preparation	3	3.2	3	3.0	
	Action	1	1.0	4	4.0	
	Maintenance	1	1.0	3	3.0	
3 months						0.002
(control: *n* = 71; intervention: *n* = 82)	Precontemplation	56	78.9	46	56.1	
	Contemplation	4	5.6	13	15.9	
	Preparation	7	9.9	2	2.4	
	Action	3	4.2	19	23.2	
	Maintenance	1	1.4	2	2.4	
6 months						0.059
(control: *n* = 68; intervention: *n* = 79)	Precontemplation	48	70.6	41	51.9	
	Contemplation	7	10.3	13	16.5	
	Preparation	6	8.8	4	5.2	
	Action	5	7.4	16	20.2	
	Maintenance	2	2.9	5	6.3	

Statistical comparisons between the control and intervention groups were performed using the Fisher’s exact probability test

No significant difference was observed in the distribution of stages of change (precontemplation, contemplation, preparation, action, and maintenance) between the two groups at baseline. The differences in the proportion of the distribution of these behavioral modification stages between the control and intervention groups were significant at 3 months (*p* < 0.001) but marginally significant at 6 months (*p* = 0.059).

## Discussion

To the best of our knowledge, this is the first study to examine the effectiveness of five types of genotypes based on the alcohol-metabolizing enzyme (i.e., *ALDH2* and *ADH1B*) and personalized brief interventions using genotype information (SBI) on alcohol consumption and drinking behavior in college students and adults aged 20–30 years. Although alcohol consumption and AUDIT-C scores did not change notably in the control group, they significantly decreased in the intervention group. Moreover, the reduction in these indicators from baseline values was significantly greater in the intervention group than in the control group. We also observed alterations in the behavioral modification stages of alcohol use by the intervention. These results suggest that a personalized brief intervention using alcohol metabolism-related genotype information is an effective strategy for reducing alcohol consumption and preventing the development of high-risk excessive drinking in young adults.

In this study, participants were assigned to two groups using a simple randomization table to eliminate any bias in the grouping. Theoretically, this randomization method is known to eliminate differences between groups for each indicator [30], and no differences, except for the sex composition ratio, were found between the groups. Although the amount of alcohol consumption was largely different between sexes [29], owing to the fact that no differences were found in terms of alcohol consumption at baseline between the groups, and that we used sex as an adjustment variable in the subsequent analysis of comparisons between groups, we believe that randomization itself was not a problem in this study. However, this study had a relatively large number of dropouts during the study period (29.2% in the control group and 21.0% in the intervention group). As described in Fig. 1, the control group had a large dropout rate at 1 month after randomization, while the intervention group had almost no dropout until the genetic test results were disclosed, after which a large dropout was observed during the follow-up period. Collectively, we speculate that the difficulty in maintaining and recording drinking calendar and answering the questionnaire caused the dropout, rather than the genetic testing or brief intervention itself. In this study, many participants used a calendar and did not forget to record their drinking behavior on drinking occasions. Nevertheless, an electronic tool that can easily convert and record the amount of alcohol consumed could be used in future studies.

However, as shown in Additional file 1:Tables S2 and S3, no differences in baseline characteristics, except for sex, were observed in the populations excluding dropouts, and the sample size for the alcohol consumption and AUDIT-C score was large enough for analysis by separating the analysis perspectives (i.e., comparing time-series within the same group and comparing the change from baseline at the same time point between the groups). Hence, the dropout of participants in this study did not appear to have a significant effect on the results.

In the intervention group, alcohol consumption and AUDIT-C scores decreased significantly at 3 and 6 months compared with those at baseline. Moreover, the reduction in alcohol consumption (at 3 months) and AUDIT-C score (at 3 and 6 months) was significantly greater in the intervention group than in the control group. These results suggest that SBI in the current study may be a useful approach for reducing alcohol consumption among young excessive drinkers. Previous studies have reported that brief intervention targeted at individual *ALDH2* genetic polymorphism significantly decreased alcohol consumption [17, 18], which supports our present findings. However, these previous studies did not compare the changes in alcohol consumption between the control and intervention groups, indicating that the effectiveness of brief interventions with genetic information was not clearly proven. Our present results strictly prove that SBI induced a greater reduction in alcohol consumption than the control condition. Previous studies have conducted SBI using only *ALDH2* genetic polymorphisms but did not use *ADH1B* genotype information [17, 18]. In contrast, participants in the intervention group received a brief intervention with genetic polymorphism information regarding both the *ALDH2* and *ADH1B* genetic polymorphisms. Moreover, the biological and genetic information on the pathogenesis of alcohol-related diseases and the rationale for prevention as an intervention using easy-to-understand pamphlets were also provided with the above feedback from the genotype screening. Thus, personalized interventions may have led to a greater reduction in alcohol consumption in this study.

Regarding the reasons for the reduction in alcohol consumption and AUDIT-C score by SBI, we focused on behavioral changes in alcohol use using a TTM [21]. Interestingly, the results showed a significant difference in the composition of the behavioral change stages between the intervention and control groups at 3 months, suggesting that the proportion of “Precontemplation” stage decreased and that of “Action” stage increased in the intervention group. Therefore, it is possible that these behavioral changes contributed to the decrease in alcohol consumption values and AUDIT-C scores in the intervention group. Although studies have reported that genetic information feedback reduces alcohol consumption [17, 18], the factors that contribute to this reduction have not been examined. Therefore, this study provides the first evidence that SBI reduces alcohol consumption, possibly through behavioral changes. Nevertheless, the extent to which the elements of SBI contribute to changes in drinking behavior is currently unclear. Future studies should address this issue in more detail.

In contrast, a significant reduction in alcohol consumption in the intervention group was attenuated from 3 to 6 months and a significantly greater reduction in alcohol consumption was observed in the intervention group compared with the control group at 3 months but not at 6 months. This result suggests that the alcohol consumption-reducing effect of SBI does not persist in the long term. A recent umbrella review concluded that cognitive behavioral therapy (CBT) and motivational interviewing (MI) show effectiveness in the relative long-term (more than 6 months) reduction of alcohol consumption [31]. Both CBT and MI are usually conducted over multiple sessions [32, 33]; reports of some cases indicate that more sessions induce a greater reduction in alcohol consumption than a single session [34]. Because the SBI was implemented only once in this study, future studies should investigate the effectiveness of multiple educational campaigns conducted on a periodic schedule (e.g., once every 3–6 months) to sustain the effectiveness of the SBI.

Additionally, despite the lack of a special intervention, the AUDIT-C score decreased from baseline to 6 months in the control group. The participants in the control group were asked to read and study the same traditional educational materials for university students as the intervention group. Generally, visualizing personal health data motivates users to increase or maintain their activities [34]. In addition, the Hawthorne effect on alcohol-use behavior may have occurred in this study [35]. Accordingly, participating in this study may have contributed to the reduction in alcohol consumption among young adults, regardless of whether genetic testing information was fed back to the participants.

In this study, we provide a new approach to reducing alcohol consumption among excessive drinkers, which is not only an academic contribution but also beneficial to public health. Considering the current situation in which individual differences in health literacy exist, with those with low health literacy in childhood maintaining the health literacy at low levels in later life [36], SBI may be effective in the workplace and among other adult age groups. It is desirable to expand the target population in the future.

This study had some limitations. First, 21 participants (21.0%) in the intervention group and 28 (29.2%) in the control group were lost to follow-up during the study period. However, a previously reported intervention reducing alcohol consumption for college students confirmed that approximately 40% participants drop out at 6 months [37], indicating that the compliance rate of our study was relatively high. In addition, the number of participants who completed the current study up to the 6-month point was similar to that reported in a previous study [28]. Hence, our sample size, excluding the dropouts, may be valid for the investigation of our research questions and might not have a significant impact on the data interpretation and conclusions of this study. Nevertheless, the possibility that some participants dropped out owing to unchanged or increased alcohol consumption cannot be denied. Thus, we may have under- or over-estimated the effects of SBI on alcohol-use behavior.

Second, data on alcohol consumption and AUDIT-C were self-reported by the participants; therefore, interpreting the results requires caution. Third, we did not compare the effect of SBI on alcohol-use behavior between the five types of alcohol-metabolizing enzyme genotypes or sexes because of the small sample size, whereas previous studies demonstrated that the responsiveness of alcohol-reducing interventions differed between the genotypes and sexes [17, 29]. In contrast, a significant reduction in alcohol consumption was observed in the intervention group, which had a relatively high proportion of women; this finding may be clinically important because alcohol-related health problems have been increased recently not only among men but also among women [38]. Finally, we cannot ignore the unmeasurable confounding factors that may have affected the results of this study. Future studies should include larger populations with better outcomes.

## Conclusions

The effectiveness of our individualized intervention for lifestyle modification, considering genetic constitution information, was confirmed by the significant decrease in average daily alcohol consumption and AUDIT-C scores for up to 6 months associated with a significant increase in the proportion of participants who transitioned from the precontemplation stage to the contemplation and action stages. This type of intervention might be an effective strategy for preventing excessive drinking behavior in young adults.

### Supplementary Information

Additional file 1: Table S1 Alcohol-use disorders identification test (AUDIT, AUDIT-C). Table S2 Baseline characteristics of the participants, excluding dropouts up to 3 months from baseline. Table S3 Baseline characteristics of the participants, excluding dropouts up to 6 months from baseline.

## Data Availability

All data required to support the protocol will be supplied on request, with the approval of the participants. Datasets generated and/or analyzed during the current study are not published and are not available, but after data collection is completed, they will be available from the corresponding author and first author upon reasonable request (in Japanese). The datasets and statistical codes analyzed during the current study as well as the full protocol are available from the corresponding authors upon reasonable request.

## Abbreviations

*ALDH2*: Aldehyde dehydrogenase 2
*ADH1B*: Alcohol dehydrogenase 1B
AUDIT: Alcohol Use Disorders Identification Test
AUDIT-C: AUDIT-Consumption
SBI: Screening and brief intervention
TTM: Transtheoretical model
