# Supplementary material for: Effectiveness of genetic feedback on alcohol metabolism to reduce alcohol consumption in young adults: an open-label randomized controlled trial
Source: BMC Med. 2024 May 20;22:205. doi: 10.1186/s12916-024-03422-y (PMC11106878; doi:10.1186/s12916-024-03422-y)
Supplement: Supplementary file 1 — Additional file 1: Table S1 Alcohol-use disorders identification test (AUDIT, AUDIT-C). Table S2 Baseline characteristics of the participants, excluding dropouts up to 3 months from baseline. Table S3 Baseline characteristics of the participants, excluding dropouts up to 6 months from baseline. [file 12916_2024_3422_MOESM1_ESM.docx]

Table S1 Alcohol use disorders identification test (AUDIT, AUDIT-C) [23,25].

| AUDIT Questions | | Scoring system | | | | |
| --- | --- | --- | --- | --- | --- | --- |
|  |  | 0 | 1 | 2 | 3 | 4 |
| AUDIT-C | How often do you have a drink containing alcohol? | Never | Monthly or less | 2 to 4 times per month | 2 to 3 times per week | 4 times or more per week |
|  | How many units of alcohol do you drink on a typical day when you are drinking? | 0 to 2 | 3 to 4 | 5 to 6 | 7 to 9 | 10 or more |
|  | How often have you had 6 or more units if female, or 8 or more if male, on a single occasion in the last year? | Never | Less than monthly | Monthly | Weekly | Daily or almost daily |
| How often during the last year have you found that you were not able to stop drinking once you had started? | | Never | Less than monthly | Monthly | Weekly | Daily or almost daily |
| How often during the last year have you failed to do what was normally expected from you because of your drinking? | | Never | Less than monthly | Monthly | Weekly | Daily or almost daily |
| How often during the last year have you needed an alcoholic drink in the morning to get yourself going after a heavy drinking session? | | Never | Less than monthly | Monthly | Weekly | Daily or almost daily |
| How often during the last year have you had a feeling of guilt or remorse after drinking? | | Never | Less than monthly | Monthly | Weekly | Daily or almost daily |
| How often during the last year have you been unable to remember what happened the night before because you had been drinking? | | Never | Less than monthly | Monthly | Weekly | Daily or almost daily |
| Have you or somebody else been injured as a result of your drinking? | | No | **－** | Yes, but not in the last year | **－** | Yes, during the last year |
| Has a relative or friend, doctor or other health worker been concerned about your drinking or suggested that you cut down? | | No | **－** | Yes, but not in the last year | **－** | Yes, during the last year |

Scoring of AUDIT: 0 to 7 indicates low risk, 8 to 14 indicates increasing risk, 15 or more indicates possible dependence in Japan [Ministry of Health, Labour and Welfare, Japan]

AUDIT-C is the first three of the ten AUDIT items.

| Variable | | Control (n = 73) | |  | Intervention (n = 84) | |  | *P*-value |
| --- | --- | --- | --- | --- | --- | --- | --- | --- |
|  |  | n | % or mean (median) |  | n | % or mean (median) |  |  |
| Age (years) | | 71 | 22.5 (21.0) |  | 83 | 22.8 (22.0) |  | 0.349^a^ |
| Female | | 52 | 73.3 |  | 40 | 47.6 |  | 0.001^b^ |
| Carrer status | |  |  |  |  |  |  | 0.983^b^ |
|  | Undergraduate student | 43 | 60.6 |  | 52 | 61.9 |  |  |
|  | Graduate student | 16 | 22.5 |  | 18 | 21.4 |  |  |
|  | Faculty and Staff | 12 | 16.9 |  | 14 | 16.7 |  |  |
| Employment | |  |  |  |  |  |  | 0.756^b^ |
|  | Not working | 8 | 11.3 |  | 12 | 14.3 |  |  |
|  | Part-time job | 52 | 73.2 |  | 57 | 67.9 |  |  |
|  | University staffs, resident,  teachers or part-time lecturers | 11 | 15.5 |  | 15 | 17.9 |  |  |
| Circle and hobby activities | |  |  |  |  |  |  | 0.963^b^ |
|  | Have | 42 | 59.2 |  | 50 | 59.5 |  |  |
|  | Not | 29 | 40.8 |  | 34 | 40.5 |  |  |
| Currently status of living | |  |  |  |  |  |  | 0.638^b^ |
|  | Living with family | 13 | 18.3 |  | 13 | 15.5 |  |  |
|  | Living alone | 58 | 81.7 |  | 71 | 84.5 |  |  |
| Classification based on alcohol-metabolizing enzyme genotype testing | |  | |  |  |  |  | 0.928^c^ |
|  | A | 4 | 5.9 |  | 3 | 3.6 |  |  |
|  | B | 51 | 75.0 |  | 65 | 77.4 |  |  |
|  | C | 1 | 1.5 |  | 1 | 1.2 |  |  |
|  | D | 12 | 17.6 |  | 15 | 17.9 |  |  |
|  | E | 0 | 0.0 |  | 0 | 0.0 |  |  |
| AUDIT-10 | | 70 | 8.0 (7.0) |  | 84 | 9.0 (7.0) |  | 0.238^a^ |
| AUDIT-C | | 71 | 5.4 (5.0) |  | 84 | 5.8 (6.0) |  | 0.282^a^ |
| Average number of drinks | | 71 | 5.4 (5.0) |  | 84 | 5.7(5.0) |  | 0.429^a^ |
| Average daily alcohol consumption (g) | | 71 | 41.8 (39.7) |  | 84 | 43.9 (38.8) |  | 0.600^a^ |

Table S2 Baseline characteristics of the participants excluding dropouts up to 3 months of the study (n = 157).

Statistical comparisons between control and intervention groups using ^a^Wilcoxon’s rank sum test. ^b^Chi-square test, ^c^Fisher’s exact probability test, respectively.

Table S3 Baseline characteristics of the participants excluding dropouts up to 6 months of the study (n = 147).

| Variable | | Control (n = 68) | |  | Intervention (n = 79) | |  | *P*-value |
| --- | --- | --- | --- | --- | --- | --- | --- | --- |
|  |  | n | % or mean (median) |  | n | % or mean (median) |  |  |
| Age (years) | | 68 | 22.6 (21.0) |  | 78 | 22.9 (22.0) |  | 0.285^a^ |
| Female | | 50 | 73.5 |  | 36 | 45.6 |  | 0.001^b^ |
| Carrer status | |  |  |  |  |  |  | 0.994^b^ |
|  | Undergraduate student | 41 | 60.3 |  | 47 | 59.5 |  |  |
|  | Graduate student | 15 | 22.1 |  | 18 | 22.8 |  |  |
|  | Faculty and Staff | 12 | 17.6 |  | 14 | 17.7 |  |  |
| Employment | |  |  |  |  |  |  | 0.710^b^ |
|  | Not working | 8 | 11.8 |  | 12 | 15.2 |  |  |
|  | Part-time job | 49 | 72.1 |  | 52 | 65.8 |  |  |
|  | University staffs, resident,  teachers or part-time lecturers | 11 | 16.2 |  | 15 | 19.0 |  |  |
| Circle and hobby activities | |  |  |  |  |  |  | 0.921^b^ |
|  | Have | 41 | 60.3 |  | 47 | 59.5 |  |  |
|  | Not | 27 | 39.7 |  | 32 | 40.5 |  |  |
| Currently status of living | |  |  |  |  |  |  | 0.673^b^ |
|  | Living with family | 13 | 19.1 |  | 13 | 16.5 |  |  |
|  | Living alone | 55 | 80.9 |  | 66 | 83.5 |  |  |
| Classification based on alcohol-  metabolizing enzyme genotype testing | | |  |  |  |  |  | 0.783^c^ |
|  | A | 4 | 5.9 |  | 2 | 2.5 |  |  |
|  | B | 51 | 75.0 |  | 61 | 77.2 |  |  |
|  | C | 1 | 1.5 |  | 1 | 1.3 |  |  |
|  | D | 12 | 17.6 |  | 15 | 19.0 |  |  |
|  | E | 0 | 0.0 |  | 0 | 0.0 |  |  |
| AUDIT | | 67 | 8.0 (7.0) |  | 79 | 8.9 (7.0) |  | 0.230^a^ |
| AUDIT-C | | 68 | 5.4 (5.0) |  | 79 | 5.7 (6.0) |  | 0.302^a^ |
| Average number of drinks | | 68 | 5.3 (5.0) |  | 79 | 5.6 (5.0) |  | 0.483^a^ |
| Average daily alcohol consumption (g) | | 68 | 42.1 (38.6) |  | 79 | 43.6 (38.1) |  | 0.777^a^ |

Statistical comparisons between control and intervention groups using ^a^Wilcoxon’s rank sum test. ^b^Chi-square test, ^c^Fisher’s exact probability test, respectively.
